# Operative Repair of Medial Patellofemoral Ligament Injury Versus Knee Bracing in Acute First-Time Traumatic Patellar Dislocation: A Systematic Review and Meta-Analysis

**DOI:** 10.7759/cureus.73984

**Published:** 2024-11-19

**Authors:** Ahmed Elnewishy, Abdelfatah M Elsenosy, Sam Nahas, Mohamed Salem, Hagar Teama

**Affiliations:** 1 Orthopaedics, Royal Berkshire Hospital, Reading, GBR; 2 Trauma and Orthopaedics, University Hospital Dorset, Poole, GBR; 3 Orthopaedics and Trauma, Royal Berkshire Hospital, Reading, GBR; 4 General Surgery, Kings Mill Hospital, Nottingham, GBR; 5 Pharmacy, Kafr El Sheikh Hospital, Kafr El Sheikh, EGY

**Keywords:** acute, acute patellar dislocation, first-time patellar dislocation, knee brace, kujala score, lysholm score, mpfl, mpfl reconstruction, operative repair, traumatic patellar dislocation

## Abstract

Acute first-time traumatic patellar dislocation is a prevalent knee injury, particularly in adolescents, often managed conservatively with knee bracing. Recently, medial patellofemoral ligament (MPFL) reconstruction has gained popularity for its potential benefits in reducing redislocation rates and enhancing functional outcomes. This systematic review and meta-analysis compared the outcomes of MPFL reconstruction versus knee bracing for managing acute first-time traumatic patellar dislocation. A comprehensive search of PubMed, Scopus, Google Scholar, and the Cochrane Library identified studies published within the last 10 years that directly compared these treatment approaches, with primary outcomes focusing on redislocation rates and functional recovery measured by Kujala scores. A total of six studies, involving 325 patients, were included in the analysis. Results indicated that MPFL reconstruction significantly reduced redislocation rates (OR: 0.17, 95% CI: 0.09 to 0.32, P < 0.00001) and improved functional outcomes (MD in Kujala scores: 8.10, 95% CI: 6.46 to 9.75) compared to knee bracing. Despite notable heterogeneity across studies (I² = 95%), MPFL reconstruction consistently demonstrated superior long-term knee stability and fewer reoperations. These findings suggest that surgical intervention is the preferred treatment for long-term stability; however, further high-quality randomized controlled trials are recommended to confirm these results.

## Introduction and background

Background

Acute first-time lateral patellar dislocation is a prevalent knee injury, especially among adolescents, and it commonly presents with acute knee hemarthrosis. The estimated incidence of first-time patellar dislocations in adolescents is approximately 29 cases per 100,000 person-years [1]. This type of dislocation can lead to several complications, including osteochondral injuries, ongoing knee pain, and limitations in physical activity.

Over the long term, these injuries contribute significantly to patellofemoral arthritis, with cumulative incidence rates of symptomatic arthritis reaching between 39% and 49% within 25 years - a high figure with important implications for patients’ joint health and quality of life [2,3]. Lateral patellar dislocations are particularly common in individuals engaged in high-impact sports, such as basketball, soccer, and gymnastics, which involve frequent jumping, twisting, or pivoting motions. Additionally, certain physically demanding occupations that require kneeling, squatting, or sudden directional changes can predispose individuals to a higher risk of this injury.

The medial patellofemoral ligament (MPFL) is a key stabilizing structure that prevents excessive lateral displacement of the patella. This ligament connects the medial side of the patella to the femur, providing critical resistance against lateral patellar dislocation, especially during knee flexion. Injuries to the MPFL can destabilize the patella, leading to recurrent dislocations, pain, and long-term patellofemoral complications. Management of MPFL injuries often includes either repair or reconstruction. MPFL repair involves reattaching the injured ligament to its anatomical location, while reconstruction entails creating a new ligament structure, usually from a tendon graft, to restore stability. These approaches have distinct implications for knee mechanics and patient recovery, and each is used based on the severity of the injury and patient-specific factors. The mechanism underlying most traumatic patellar dislocations involves a twisting motion of the knee while the foot remains firmly planted. Several anatomical factors predispose individuals to this injury, such as trochlear dysplasia, patella alta, and an increased tibial tubercle (TT) to trochlear groove (TG) distance. These factors, along with trauma, often lead to repeated dislocations, further complicating patient outcomes. Additionally, lateral patellar dislocations are more common in females, potentially due to anatomical differences such as a wider pelvis and increased Q-angle, which may predispose them to instability [4,5].

Diagnosis typically involves a thorough physical examination and is supported by imaging studies like magnetic resonance imaging (MRI). MRI findings that support a diagnosis of patellar dislocation often include injury to the MPFL and bone edema on the medial patellar facet and lateral femoral condyle, further guiding clinical management decisions [6].

When considering treatment for first-time patellar dislocation, some clinicians recommend surgical interventions to stabilize the patella and prevent future dislocations. Options include medial reefing repair [7], MPFL repair [8], and MPFL reconstruction [9], each demonstrating success in reducing redislocation rates. A recent meta-analysis by Previtali et al. [10] suggests that MPFL repair and other soft tissue techniques in the medial patellofemoral complex effectively reduce redislocation in patients with recurrent patellar instability. However, the choice of intervention for initial dislocations remains a subject of ongoing debate among clinicians.

In recent years, there has been a noticeable shift toward surgical MPFL stabilization in younger populations. Evidence indicates that surgical treatment, particularly in children and adolescents, is often more effective than nonoperative approaches in reducing redislocation, improving quality of life, and enhancing athletic performance [8,11]. Conversely, non-anatomic methods for patellar stabilization may lower the likelihood of redislocation but are often associated with a greater risk of degenerative changes in the joint, which can negatively impact knee function in the long term [12,13].

**Figure 1:**
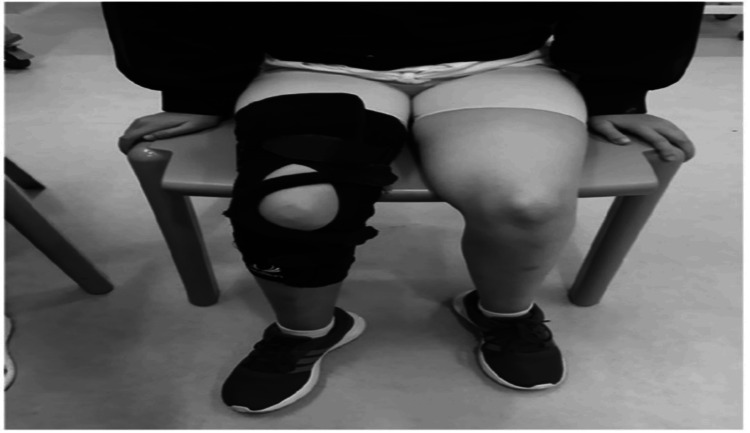
Example of a patella-stabilizing brace that can be used for recovery following a first-time dislocation.

Nonetheless, non-surgical treatment is frequently considered the first-line approach, especially in younger patients. Factors influencing the decision for non-surgical intervention include lower severity of initial injury, absence of anatomical abnormalities, and the desire to avoid surgical risks. Elastic knee braces, for instance, aid recovery by facilitating weight-bearing, easing patient anxiety, and promoting patellar realignment, as illustrated in Figure 1 [14].

In recent years, the debate over whether surgical or conservative treatment is more effective for managing first-time patellar dislocations has intensified [15,16]. A notable Cochrane review concluded that while there is some evidence suggesting surgery may offer better short-term outcomes than non-surgical approaches, the overall quality of this evidence is low, primarily due to bias and inaccuracies in effect estimation [17]. Although surgical interventions typically show a reduction in redislocation rates, their impact on long-term functional outcomes remains ambiguous, as highlighted by several studies [18].

Given these ongoing uncertainties, we conducted this systematic review to thoroughly compare the outcomes of surgical repair of the MPFL injury against conservative management using knee bracing in patients experiencing acute first-time traumatic patellar dislocation. This review meticulously evaluates critical aspects such as redislocation rates, functional recovery, and patient satisfaction, aiming to provide clear guidance based on comprehensive evidence.

Objective

This review aims to systematically evaluate and compare the clinical outcomes of operative repair via MPFL reconstruction versus conservative treatment with knee bracing in patients experiencing acute first-time traumatic patellar dislocation. The focus is on assessing the differences in redislocation rates and functional recovery between these treatment modalities.

Methods

Search Strategy

In September 2024, a comprehensive search was conducted across several databases, including PubMed, Scopus, Google Scholar, and the Cochrane Library, to identify publications comparing surgical and conservative treatments for acute first-time traumatic patellar dislocation. Search terms combined MeSH and free-text keywords such as "patellar dislocation," "MPFL reconstruction," "knee bracing," and "functional outcomes," refined with Boolean operators and filters to restrict articles to those published in English within the last decade. Reference lists of identified articles were also reviewed to uncover additional studies.

Inclusion Criteria

Eligible studies included randomized controlled trials (RCTs), cohort studies, case series, and observational studies comparing MPFL reconstruction to conservative treatments like knee bracing. Relevant studies needed to report on primary outcomes such as redislocation rates and functional recovery, quantified using the Kujala score. Only English-language articles were considered.

Exclusion Criteria

Excluded were studies that lacked a direct comparison between the two treatment approaches, as well as case reports, opinion pieces, editorials, and any lacking sufficient outcome data or not published in English.

Outcome Measures

Primary outcomes assessed included redislocation rates and functional recovery, primarily measured by the Kujala score. Secondary outcomes were patient satisfaction, reoperation rates, and other complications, providing insights into the efficacy and safety of the treatment options.

Data Extraction and Quality Assessment

Data were extracted independently by two reviewers using a standardized form to ensure consistency in capturing study characteristics, patient demographics, interventions, outcomes, and follow-up durations. Discrepancies were resolved through consensus or by consulting a third reviewer. The quality of the studies was assessed using the GRADE framework, evaluating the risk of bias, consistency, directness, and precision, and rating each study as high, moderate, low, or very low quality.

Statistical Analysis

Analysis was conducted using RevMan 5.4 (The Cochrane Collaboration, Copenhagen). Continuous outcomes like the Kujala scores were expressed as mean differences (MDs) with 95% confidence intervals (CIs), while dichotomous outcomes such as redislocation rates used odds ratios (ORs). Heterogeneity was quantified using the I² statistic; values over 50% prompted the use of a random-effects model. Publication bias was evaluated through funnel plots and Egger's test, with significance set at P <0.05.

Results

Search and Study Selection

**Figure 2:**
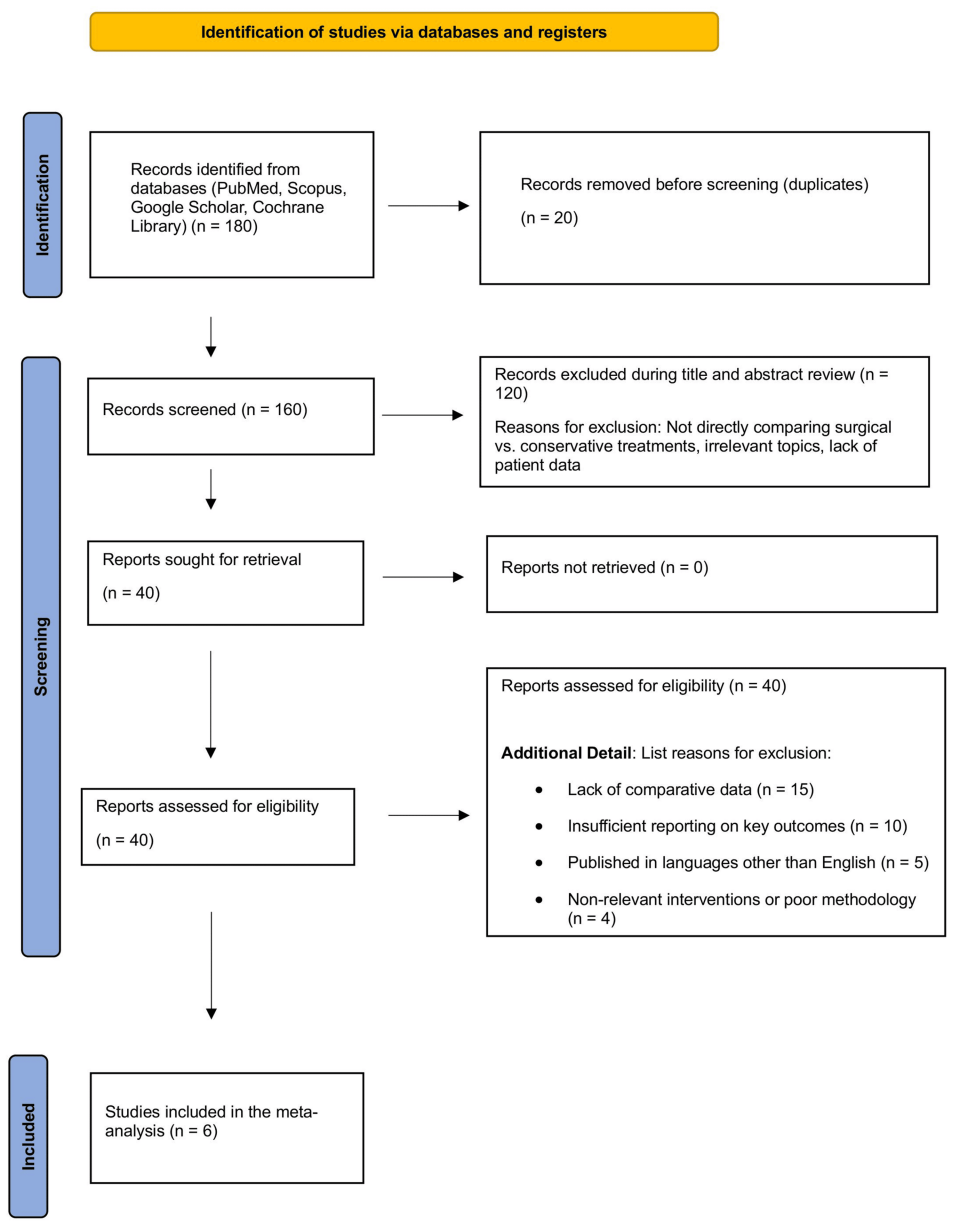
Prisma flowchart of the reviewed studies. PRISMA flow diagram illustrating the study selection process. A total of 180 records were identified from four databases (PubMed, Scopus, Google Scholar, and Cochrane Library). After removing 20 duplicate records, 160 unique records were screened. Of these, 120 were excluded during title and abstract screening for not directly comparing surgical versus conservative treatments or other irrelevant criteria. Forty full-text articles were reviewed, with 34 excluded for reasons such as lack of comparative data (n = 15), insufficient outcome reporting (n = 10), non-English language (n = 5), and non-relevant interventions or poor methodology (n = 4). Six studies were included in the final meta-analysis.

Our comprehensive search across several databases initially yielded 180 records. After careful removal of duplicates, we proceeded with 160 articles for a more detailed evaluation. Title and abstract screening led to the exclusion of 120 studies that did not meet the specific criteria of directly comparing surgical to conservative treatments for acute first-time traumatic patellar dislocation. This process narrowed the selection to 40 full-text articles, which underwent a thorough examination. The subsequent detailed review excluded 34 articles due to a lack of direct comparisons, inadequate reporting on essential outcomes like redislocation rates or functional recovery, or because they were not published in English. Furthermore, some studies were excluded due to poor methodological quality or irrelevance to the specified interventions, leaving six studies that satisfied all inclusion criteria. These studies were incorporated into the final quantitative synthesis, visually summarized in the Prisma flowchart shown in Figure 2.

Study Characteristics

The meta-analysis incorporated six studies, varying in design from RCTs to prospective and retrospective analyses. Sample sizes ranged from 36 to 76 participants, totaling 325 individuals divided into 153 in the surgical group and 172 in the conservative group. The patient demographic primarily included adolescents and young adults experiencing their first traumatic patellar dislocation, with some studies also involving skeletally mature individuals.

The interventions compared included MPFL reconstruction for the surgical group versus knee bracing for conservative management. Follow-up durations varied significantly, from one to six years, providing a robust analysis of both short- and long-term outcomes. Primary outcomes assessed were redislocation rates and functional scores, including the Kujala score and the International Knee Documentation Committee (IKDC) score, supplemented by measures of patient satisfaction.

Data Synthesis and Study Findings

Our analysis demonstrated varied results across the studies, detailed in Table 1. Notably, studies like those by Askenberger et al. and Zheng et al. highlighted significant differences in redislocation rates and functional outcomes between the groups, underscoring the effectiveness of surgical interventions over conservative methods in managing acute patellar dislocations.

**Table 1:** Data extraction table for the reviewed studies MPFL, medial patellofemoral ligament.

Category	Askenberger et al. [8]	Gurusamy et al. [19]	Ji et al. [20]	Lee and Yau [21]	Regalado et al. [22]	Zheng et al. [23]
Study Design	Randomized controlled trial	Case series (Level 4)	Randomized controlled trial	Retrospective study	Prospective randomized trial	Prospective controlled trial
Sample Size	74 patients	76 patients	62 patients	41 patients	36 patients	69 patients
Level of Evidence	Level 1	Level 4	Level 1	Level 3	Level 1	Level 2
Patient Demographics	Children aged 9-14	Adolescents 9-19 years old with loose bodies	Patients with acute patellar dislocation and MPFL injury	Patients with patellar dislocation, mean age 23.6	Adolescents 8-16 years old	Skeletally mature patients aged 15-26
Intervention Details	Knee brace (n=37) vs arthroscopic MPFL repair (n=37)	MPFL reconstruction (n=30) vs no treatment/MPFL repair (n=46)	Surgical vs nonsurgical treatment of MPFL injury	Conservative treatment vs MPFL repair	Non-surgical (n=20) vs surgical (n=16)	Surgical MPFL reconstruction (n=30) vs non-surgical treatment (n=39)
Follow-up Duration	2 years	2.6 years	42 months	1 year	6 years	2 years
Outcome Measures	Kujala score, redislocation rate, KOOS-Child score, patient satisfaction	Kujala score, recurrent instability, return to sport	Kujala score, patellar tilt, lateral shift, redislocation rate	IKDC score, redislocation rate, Tegner score	Redislocation rate, reoperation, satisfaction	Kujala score, redislocation rate, additional surgeries
Results	Redislocation rate: 43% (knee brace) vs 22% (MPFL repair); Kujala: 95.9±7.2 (brace) vs 90.9±13.0 (repair)	Recurrent instability: 58.7% (no treatment/repair) vs 10.0% (MPFL reconstruction); return to sport: 39.1% (repair) vs 66.7% (reconstruction)	Kujala score: 93.57 (surgical) vs. 80.19 (nonsurgical); redislocation: 3.3% (surgical) vs 11.5% (nonsurgical)	Conservative group recurrent dislocation: 33%; MPFL group: 0%	Redislocation at 6 years: 73% (conservative) vs 33% (operative)	Kujala score: 86.27 (surgical) vs 80.03 (nonsurgical); redislocation rate: 0% (surgical) vs. 20.5% (nonsurgical)

Quality Assessment

Each study's quality was rigorously assessed using the GRADE framework, focusing on the risk of bias, consistency, directness, and precision. These evaluations are crucial in understanding the strength of the evidence presented and are summarized in Table 2. The GRADE assessments helped confirm the reliability of the results, indicating that findings from well-conducted studies significantly support the superior efficacy of surgical interventions for preventing redislocations and enhancing knee functionality.

**Table 2:** Quality assessment of studies using the GRADE framework

Study	Study Design	Risk of Bias	Inconsistency	Indirectness	Imprecision	Publication Bias	Overall Quality
Askenberger et al. [8]	Randomized controlled trial	Low	Low	Low	Low	Low	High
Gurusamy et al. [19]	Case series	High	N/A	Moderate	High	High	Low
Ji et al. [20]	Randomized controlled trial	Low	Low	Low	Low	Low	High
Lee and Yau [21]	Retrospective cohort study	Moderate	N/A	Moderate	Moderate	Moderate	Moderate
Regalado et al. [22]	Randomized controlled trial	Low	Low	Low	Low	Low	High
Zheng et al. [23]	Prospective controlled trial	Moderate	N/A	Moderate	Moderate	Moderate	Moderate

Results of meta-analysis

Functional Outcomes (Kujala Scores)

**Figure 3:**
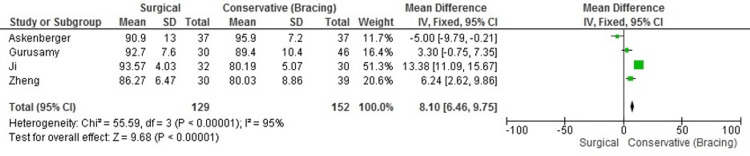
Forest plot of Kujala scores showing functional outcome differences between surgical and conservative treatments. Data sourced from Refs. [8,19,20,23].

The pooled analysis of functional outcomes, specifically using the Kujala score, revealed significant improvement in knee function among patients undergoing surgical intervention compared to those treated conservatively. The Kujala score is a patient-reported outcome measure specifically designed to assess anterior knee pain and function. It evaluates various aspects of knee functionality, including pain, limp, and ability to perform daily activities, making it an appropriate tool for assessing recovery in patients with patellar dislocations. The MD in Kujala scores between groups was 8.10 (95% CI: 6.46 to 9.75). This finding indicates a clear benefit in favor of surgery, with patients generally reporting better functional outcomes post-surgery. However, the data displayed considerable heterogeneity, with an I² value of 95%. This substantial variability suggests that differences in study design, patient populations, and follow-up durations likely influenced the outcomes. Despite these variations, the consensus across the studies supports a more favorable outcome for surgical intervention in terms of knee functionality after acute patellar dislocation (Figure 3).

**Figure 4:**
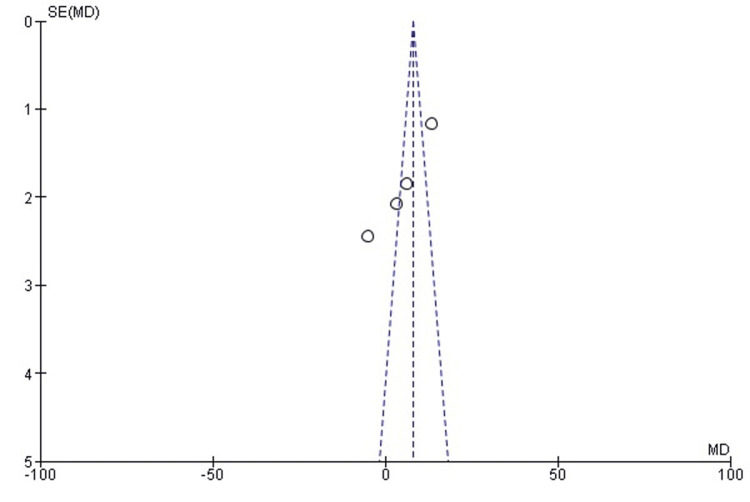
Funnel plot assessing publication bias for functional outcomes (Kujala scores) in the included studies. MD, mean difference.

Publication bias for functional outcomes (Kujala scores):* *Examination of publication bias through a funnel plot indicated a symmetrical distribution of studies around the pooled mean effect size, suggesting minimal publication bias. This observation was confirmed by Egger's test, which yielded a P-value of 0.994, reinforcing the absence of significant publication bias in the reporting of functional outcomes (Figure 4).

Redislocation Rates

**Figure 5:**
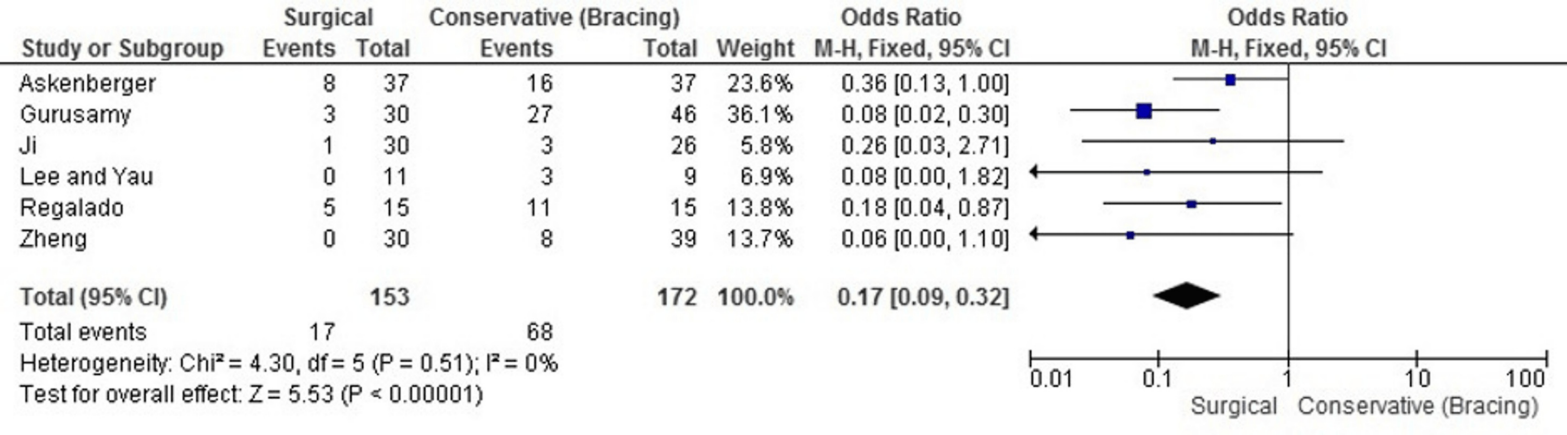
Forest plot comparing redislocation rates between MPFL reconstruction and knee bracing. MPFL, medial patellofemoral ligament. Data sourced from Refs. [8,19-23].

Our meta-analysis robustly demonstrated that surgical treatment significantly reduces the risk of redislocation when compared with conservative management. The pooled OR was 0.17 (95% CI: 0.09 to 0.32, P < 0.00001), indicating an 83% reduction in the relative risk of experiencing a redislocation among those who underwent surgical procedures. Notably, there was no observed heterogeneity among the included studies (I² = 0%), suggesting that the results are consistent and reliable across different research contexts. This uniform effectiveness highlights MPFL reconstruction as a particularly effective intervention for preventing redislocation in patients with first-time traumatic patellar dislocation (Figure 5).

**Figure 6:**
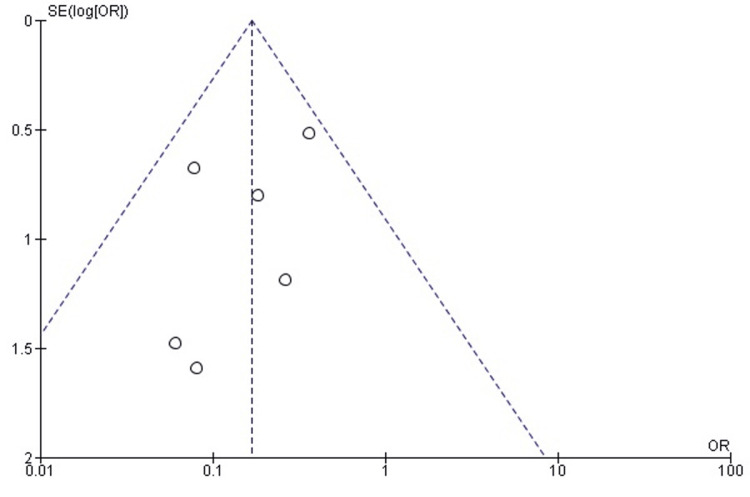
Funnel plot assessing publication bias for redislocation rates in the included studies.

Publication bias for redislocation rates: The funnel plot for assessing publication bias in the measurement of redislocation rates showed a symmetrical spread of the studies, with a corresponding Egger's test p-value of 0.178. This result implies a lack of significant publication bias, supporting the robustness and reliability of the meta-analytical findings on redislocation rates (Figure 6).

## Review

Discussion

Acute patellar dislocations, particularly first-time incidents, pose a complex challenge in sports medicine and orthopedics, as they often lead to knee instability, recurrent dislocations, and long-term patellofemoral joint complications. Management strategies for patellar dislocation include conservative approaches, such as knee bracing, and surgical interventions, notably MPFL reconstruction. MPFL reconstruction aims to stabilize the patella by reconstructing the ligament that restrains lateral movement, while knee bracing provides external support to prevent further dislocation without modifying underlying anatomical factors. Given the potential for recurrent instability with non-operative treatment, recent studies have focused on the effectiveness of MPFL reconstruction in reducing redislocation rates and improving functional outcomes, as measured by patient-reported scoring systems such as the Kujala and Lysholm scores. Comparisons with international studies reveal similar benefits of MPFL reconstruction, consistently showing lower redislocation rates and improved patient outcomes. Studies such as those by Sillanpää et al. and Arendt et al. [24,25] confirm MPFL reconstruction's superior outcomes compared to conservative treatments, reinforcing its efficacy for reducing dislocation recurrence and enhancing knee stability over time.

Our meta-analysis indicates that MPFL reconstruction has distinct advantages over knee bracing, primarily in functional outcomes and stability. For example, the findings align with previous studies, including a systematic review by Sillanpää et al., which compared operative and non-operative treatments in adolescents. Sillanpää et al. [24] found that MPFL reconstruction substantially lowered redislocation rates and improved subjective knee function compared to conservative treatment, reinforcing the potential for reconstruction to address patellar instability more effectively over time. Another study by Arendt et al. [25] on young, active adults with primary patellar dislocation found that MPFL reconstruction was associated with greater joint stability and a faster return to sport than bracing, echoing the outcomes seen in our analysis. These comparisons suggest that MPFL reconstruction may yield better clinical outcomes across different age groups and activity levels, particularly when patellar instability is a primary concern.

Notably, the redislocation rates reported in various studies consistently favor MPFL reconstruction over knee bracing. For instance, Schlumberger et al. [26] reported a redislocation rate of only 6.7% in skeletally immature patients undergoing MPFL reconstruction, compared to higher rates observed in conservative treatments. Similarly, Boelch et al. [27] documented a redislocation rate of 5.6% following MPFL reconstruction over a five-year follow-up, which contrasts sharply with redislocation rates in conservative treatments that can exceed 30% in some studies, particularly when no secondary stabilizing procedure is performed. Our findings also parallel those of Heo et al. [28], who, in their systematic review, observed that MPFL repair, while beneficial, did not achieve the same level of redislocation control as MPFL reconstruction, suggesting that reconstruction offers a more robust solution for patients requiring higher stability levels.

Further supporting the advantages of MPFL reconstruction, Tian et al. [29] conducted a meta-analysis comparing different fixation techniques within MPFL reconstruction, such as suture anchor and double transpatellar tunnel fixation. Both techniques achieved comparable redislocation rates of approximately 3%, underscoring that various surgical methods within MPFL reconstruction are effective for patellar stabilization. This consistency across techniques suggests that MPFL reconstruction's benefits are likely due to its anatomical restoration of the MPFL rather than specific surgical nuances. Moreover, in studies where patients had anatomical risk factors like trochlear dysplasia or patella alta, additional procedures combined with MPFL reconstruction were often recommended to further mitigate redislocation risk, as highlighted by Boelch et al. [27]. These findings indicate that while MPFL reconstruction is broadly effective, individualized approaches may be warranted based on patient-specific anatomy.

Limitations

This study has several limitations to consider. High heterogeneity across the included studies, particularly in patient populations, follow-up durations, and surgical techniques, affects pooled estimates and limits generalizability. Small sample sizes and a focus on short- to medium-term outcomes also reduce the statistical power and applicability of findings to longer-term scenarios. Additionally, inconsistent reporting of secondary outcomes, such as pain, satisfaction, and return to activity, hampers a comprehensive assessment. Variations in age and anatomical factors, along with potential biases, further impact comparability. These limitations highlight the need for more standardized, high-quality research for informed clinical decisions in patellar dislocation management.

## Conclusions

The results of this systematic review indicate that MPFL reconstruction provides a significant reduction in redislocation rates and improved functional outcomes compared to knee bracing for patients with acute first-time traumatic patellar dislocation. MPFL reconstruction demonstrates better long-term knee stability, making it a strong treatment option for patients who require patellar stabilization. This procedure may be particularly beneficial for younger, active patients and those with anatomical predispositions to instability, as it supports sustained joint function and reduces the likelihood of recurrent dislocations. Given these findings, MPFL reconstruction should be carefully considered, especially for individuals where long-term joint stability and function are priorities.
